# Trajectories of pain in adolescents: A prospective cohort study

**DOI:** 10.1016/j.pain.2010.09.006

**Published:** 2011-01

**Authors:** Kate M. Dunn, Kelvin P. Jordan, Lloyd Mancl, Mark T. Drangsholt, Linda Le Resche

**Affiliations:** a Arthritis Research UK Primary Care Centre, Keele University, UK; b Department of Dental Public Health Sciences, University of Washington, School of Dentistry, Seattle, WA, USA; c Department of Oral Medicine, University of Washington, School of Dentistry, Seattle, WA, USA

**Keywords:** Headache, Back pain, Stomach pain, Facial pain, Natural course

## Abstract

Identification of different patterns of change in pain over time – trajectories – has the potential to provide new information on the course of pain. Describing trajectories among adolescents would improve understanding of how pain conditions can develop. This prospective cohort study identified distinct trajectories of pain among adolescents (11–14 years) in the general population (*n* = 1336). Latent class growth analysis was carried out on the self-reported frequency of back pain, headache, stomach pain and facial pain, which was collected every 3 months for 3 years. Forty four percent of adolescents had a ‘painful’ trajectory for at least one pain site, and 12% reported persistent pain at one or more pain site. Headache was the most common; 25% of subjects were in a ‘painful’ trajectory and 5% reported persistent pain. Back pain and stomach pain were also common, with 22% and 21% of subjects in painful trajectories, respectively. Facial pain was the least common, with only 10% in a painful trajectory, and 1% reporting persistent pain. Trajectory characteristics were similar at baseline across pain sites, with the more painful trajectories having significantly higher levels of depression and somatization, lower life satisfaction and more females. Trajectories did not differ significantly at baseline in physical activity levels or BMI. Agreement of trajectory membership among pain sites was moderate. In summary, reporting a painful trajectory was common among adolescents, but persistent pain was reported by a small minority, and was usually experienced at a single pain site.

## 1 Introduction

Aches and pains are common at all ages. The prevalence of back pain ranges from up to 45% in adolescents [14], to 25% in the elderly [12], with prevalence between 20% and 30% throughout adulthood [8]. Similarly, headache prevalence ranges from up to 51% in children/adolescents, to 46% in adults and 42% in the elderly [36]. Temporomandibular pain is reported by 22–44% of adults [5,10,22], but is less common in adolescents at around 4% [27]. Stomach pain is also present in up to 20% of adolescents [33], and is a common complaint among adults [1]. These figures illustrate that episodes of pain are not simply experienced in adulthood, and suggest that the tendency for experiencing symptoms may develop in childhood or adolescence. Studies also show that symptoms persist in over a third of adolescents with pain [25,30]. Understanding the development of pain conditions over time in younger populations may provide clues to why some people experience pain throughout their lives.

Recent interest in latent class and model-based cluster analyses have facilitated the identification of typologies of variation over time, or ‘trajectories’ [32]. This parallels a rise in interest in life course epidemiology, which emphasises how trajectories can improve understanding of changes over time [18]. Modelling trajectories has advantages over simpler approaches of defining outcome at single time points, as trajectories are able to better describe the recurrent and fluctuating nature of many painful conditions than more traditional epidemiological methods. Furthermore, using trajectories, subgroups (clusters) of individuals with similar patterns of change are identified. Such methods have recently been applied to the study of adults with back pain [9]. Few studies in adolescents have collected regular data on the presence of pain in the same individuals. One study investigated changes in headache, neck and upper back pain prevalence [11]. Another study of adolescents identified trajectories of recurrent headache, stomach ache and back ache using data collected every 2 years [35]. They found that sex and anxiety/depression were the main predictors of pain trajectories. The authors concluded that further studies were needed, particularly using narrower sampling windows (i.e. more frequent measurements). Studying pain trajectories in adolescents in more detail could better describe the course of pain, and the predictors of that course. This could elucidate the beginnings of common long-term pain conditions.

Identifying trajectories of a range of different pain conditions could also ascertain similarities in trajectories between pain conditions, or overlaps of trajectory membership between different conditions. This is important, as it has been argued that there are many epidemiological similarities between different symptom-based conditions such as pain conditions [16,41]. At present it is unclear why one person gets one pain condition, and another person gets a different condition, given similar predictors across conditions. The tendency to experience one type of pain rather than another may be set early in life. The aim of this study was therefore to identify groups of adolescents defined by their trajectories of back pain, headache, stomach pain and facial pain over time, and to investigate the characteristics and overlap of trajectory membership.

## 2 Methods

The study sample, data collection procedures and measures have been described in detail elsewhere [20], and are summarized here. All procedures were approved by the Institutional Review Boards of Group Health and the University of Washington.

### 2.1 Study sample

Subjects in this cohort study were boys and girls, initially 11 years old, randomly selected from the enrollees of Group Health, a large non-profit integrated health care system in Washington State, USA. During the 1-year study recruitment period, children were sampled from the Group Health enrollment database each month. Monthly samples consisted of all enrollees (except those previously sampled) who lived in the local area and were aged from 11 years 0 months to 11 years 10 months. If multiple children meeting the age criteria lived in the same household, one child was chosen at random to participate. Children not sufficiently proficient in English to understand the interview questions, or whose parents were not sufficiently proficient in English to provide informed consent were considered ineligible.

### 2.2 Data collection procedures

An advance letter was sent to the parents of the selected children; the packet contained a fact sheet explaining the study procedures and a separate letter to the child. Parents and children who did not wish to be contacted about the study could telephone the study office to refuse participation. Households not refusing initial contact were telephoned by a survey interviewer, who spoke with the child’s parent or legal guardian and explained the study procedures in detail. Both the parent or legal guardian and the child were required to provide informed consent (adults)/assent (children) in order for the child to participate in the study.

Data on history and presence of back pain, headache, facial pain and stomach pain in the past 3 months, as well as information on demographics and suspected risk factors were collected from the child through a telephone survey lasting 25–30 min at baseline. Subjects received a $5 video store gift certificate for completing the baseline interview.

Three months after the baseline interview, and every 3 months for the next 3 years (i.e. at months 3, 6, 9, 12, 15, 18, 21, 24, 27, 30 and 33), the child received a brief mailed questionnaire with a prepaid return envelope. The questionnaire asked about the presence of each of the four pain conditions in the past 3 months. A limited number of mutable risk factors were also reassessed. Subjects who did not respond to a questionnaire were reminded by telephone and sent a new questionnaire if needed. Subjects were compensated $5 for completing each questionnaire. Those who actively refused further participation, who could not be located through health plan records or the post office (forwarding addresses) or who failed to respond to two questionnaires in a row despite reminders were not sent further questionnaires. All subjects who had not actively refused further participation were contacted for a final 3-year telephone follow-up. Over 90% of those eligible responded to this final telephone follow-up interview, which was virtually identical to the baseline interview. Subjects were compensated $10 for their participation in this final follow-up.

### 2.3 Measures

The child’s age and sex were obtained from Group Health’s enrollment database and confirmed with both the parent and the child. Data on the child’s race was gathered directly from the child.

At baseline, children were asked if they had ever had a problem with each of four pain conditions: back pain, headache, facial pain (“pain in any of the following places: the muscles of the face, the joint in front of the ear or inside the ear, other than an ear infection”), and stomach pain. They were asked to report only pain that had lasted a whole day or more, or that had occurred several times in a year. Subjects were specifically instructed not to report “little aches and pains that didn’t last very long, like a short headache or sore muscles after exercising.” Children who reported having experienced a given pain problem were reminded of the severity criteria and asked to report whether the pain problem had occurred in the past 3 months. Subjects reporting a particular pain problem were asked questions concerning pain severity, as well as pain persistence, specifically whether pain was present “almost every day,” “more than half the days” or “fewer than half the days” in the past 3 months.

In addition to questions on pain problems, the baseline interview included standardized questions on physical activity level [17] and life satisfaction [3]. Subjects reported height and weight, which were converted to Body Mass Index (BMI). Depression and somatization were assessed using abbreviated scales derived from the respective scales of the SCL-90 [7]. The SCL-90 was originally designed for adults and older adolescents (norms are provided for those aged 13 years and above [6]), but has been used successfully in studies of children as young as 11 years old [26,34]. Data from the adult population of the same health care system [39] were used to identify 6 depression items and 5 non-pain-related somatization items from the original SCL-90 scales that showed high correlations with the respective full scale (0.95 and 0.96, respectively), and adequate internal consistency (0.81 and 0.75, respectively). Mean item scores (0-low to 4-high) were calculated for each scale. Subjects were defined as having significant depressive or somatic symptoms if they scored above the 90th percentile in the entire sample (1.7 for depression and 1.2 for somatization) [21]. These factors have all been associated with pain occurrence previously, in either adolescents or adults (e.g. [2,4,15]). Pubertal development was measured using the Pubertal Development Scale (PDS) [31]. Characteristics used to assess development include height growth spurts, skin changes and body hair, plus breast development and menarche in girls, and voice changes and growth of facial hair in boys. The score is calculated from the average of the items, and ranges from 1 (development has not begun on for any of pubertal indicators) to 4 (development complete for all pubertal indicators). This measure has been shown to be acceptable, have reasonable internal consistency, and correlate well with physician ratings of pubertal development. Further details are given in a previous publication [21].

The baseline questions concerning pain problems in the past 3 months were repeated in the questionnaires mailed every 3 months.

### 2.4 Statistical analysis

Participants who returned a baseline questionnaire, consented to follow-up, and completed at least eight of the eleven follow-up questionnaires during the 33 months following baseline (3–33 months) were included in the analysis. Back pain, headache, stomach pain and facial pain were analysed separately. At each data collection point, subjects were grouped into two categories by pain frequency: (1) pain on more than half the days in the last 3 months (including almost every day), (2) pain on less than half the days in the last 3 months or no pain in last 3 months, at each follow-up time point. This dichotomy was chosen as the measures used were very sensitive, and we wished to avoid classifying subjects with occasional or transient pain as having problematic pain.

Latent Growth Curve Analysis (LCGA) was carried out to classify groups of subjects based on the trajectory of their pain frequency from 3 months to 33-months. LCGA takes into account the time-order of the outcomes (i.e. pain frequency) by applying trend structures on each cluster trajectory. Quadratic growth curves were applied for all clusters within the LCGA model. There is no definitive method of deciding on the most appropriate number of clusters (groups) and hence several goodness of fit statistics exist. We used Akaike’s information criterion (AIC), Bayes information criterion (BIC), and the Consistent AIC (CAIC) [28]. For each of these, the model with the lowest goodness of fit value indicates the optimal number of clusters. We also used bootstrap *p*-values from 500 replications to assess whether adding one extra cluster improved the model fit based on the log-likelihood (LL). The final decision on the optimal number of clusters was determined by a combination of statistical information, the size and distinctiveness of the clusters, and how well the pain profile of subjects within each cluster matched that for the cluster as a whole. Subjects are allocated to the cluster for which they have the highest posterior probability of belonging. This technique means that people with some missing data can be included. However, inclusion of people with more missing data can mean that cluster allocation is less reliable. Therefore, we chose a cut point of including people with data on at least two thirds of the follow-up points. There were no patterns of missingness apparent in the included subjects. Cluster-specific probabilities of reporting of pain allow profiles of the trajectory of pain to be developed for each cluster. Analyses were performed using LatentGold 4.0 [37].

The derived clusters were compared at baseline and 3-year follow-up with respect to gender, somatization, depression, life dissatisfaction, physical activity and BMI. Differences between groups were assessed using one-way ANOVA and chi-squared tests as appropriate.

Agreement beyond chance between trajectory membership for each pain site was compared for individuals to examine whether, for example, membership in a ‘no pain problem’, ‘painful’ or ‘persistent pain’ cluster for one pain was linked to membership in a similar cluster for another pain site. Cohen’s kappa was used to quantify agreement [19].

## 3 Results

### 3.1 Response

At baseline, 1996 subjects (49%) completed the interview. Baseline interview data for these subjects has previously been reported [21]. As described in that publication, response rate did not differ by gender or age and the sample was racially similar to the underlying population of the health care system. It was also demographically similar to the population of the greater Seattle, Washington metropolitan area.

The number of subjects who had complete data for a specific pain condition at eight or more of the 11 monthly data collection points ranged from 1333 to 1336 subjects, depending on the pain problem. Between 1183 and 1283 subjects provided data each month. Included subjects with eight or more monthly questionnaires were more likely to be female than those returning fewer questionnaires (53% vs. 43%), and were less likely to have ever had each of the pain conditions, e.g. 23% of included subjects said they had experienced back pain, compared to 29% of non-included subjects. Subjects included in the analysis also had slightly lower mean baseline depression and somatization scores than those not included (0.66 vs. 0.83 and 0.47 vs. 0.58, respectively). A similar proportion of the included and non-included subjects (83% vs. 86%) reported engaging in strenuous physical activity in the year prior to baseline.

### 3.2 Trajectories

For all pain conditions, at least 90% of subjects had a probability of over 70% of belonging to the cluster to which they were allocated, indicating that the majority of subjects were clearly allocated to one cluster.

### 3.3 Back pain trajectories

**Fig. 1:**
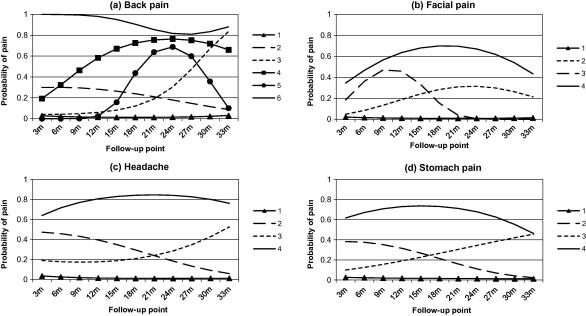
Trajectories of pain over time by pain site.

Based on the goodness of fit statistics (Table 1), the size and distinctiveness of clusters, and how well subjects matched their allocated cluster, the six-cluster solution was identified as the most appropriate for back pain. The trajectories of the identified clusters are presented in Fig. 1a, and their baseline characteristics are presented in Table 2. The largest cluster (cluster 1; 78%) had a very low probability of pain throughout follow-up, and may be characterised as a ‘no pain problem’ cluster. Two clusters had increasing probability of pain throughout follow-up. The cluster with an early pattern of increase (cluster 4, 4%) had a high proportion of females (83%) and high somatization and depression scores at baseline. The cluster with a later increase (cluster 3, 4%) were more satisfied with their life, and had moderate baseline somatization and depression scores. One cluster had low probability of pain at the beginning and end of follow-up, but a peak at around 2 years (cluster 5, 2%). The second most common group, (cluster 2, 10%) had a relatively low and decreasing probability of back pain during follow-up, and had the highest proportion of males apart from the no pain problem cluster; less than a third of this cluster were very satisfied with their life at baseline. The smallest cluster with only 1.3% of the sample had very high probability of pain throughout follow-up (cluster 6). This group had the highest proportion of females, the highest somatization and depression scores at baseline, had the highest PDS score, and were the least likely to be satisfied with their life of any cluster identified at any site.

### 3.4 Facial pain trajectories

LCGA analysis and assessment of the clusters suggested a four cluster solution as being the optimal model (presented in Fig. 1b). Facial pain was the least common pain condition reported; 90% of the sample was grouped into a ‘no pain problem’ cluster (cluster 1). The next most common cluster (6%) had a trajectory showing increased pain probability during follow-up, peaking around 2 years (cluster 2). This group had the highest proportion of males of any cluster identified (49%), and the highest mean PDS score (Table 2). Cluster 3 (2%) had increasing probability of pain during the first 9 months of follow-up, which fell to virtually zero by 21 months; over three-quarters of this cluster were female. The cluster with the highest probability of facial pain throughout follow-up (cluster 4, 1% of sample) had the worst baseline somatization and depression scores of any of the clusters at any pain site, and also had low probability of being satisfied with their life at baseline.

### 3.5 Headache trajectories

LCGA analysis on the headache data identified a four cluster solution as optimal (see Fig. 1c). The largest cluster (75%) had very low probability of headache throughout follow-up, and may be characterised as a ‘no pain problem’ cluster. Headache was the most common pain site, as a quarter of the sample was grouped into ‘painful’ trajectories. Eleven percent of the cohort fell into cluster 2, which had a decreasing probability of headache during follow-up. As with the similar decreasing cluster in back pain, this group had the highest proportion of males of all except the no pain problem cluster (Table 2). One cluster had probability of headache rising after the 18 month follow-up. This cluster (8%) had slightly lower baseline somatization and depression than clusters 2 and 4, but had the highest PDS score. Cluster 4 (5%) had high probability of pain throughout follow-up, with a peak at around 2 years (age 13). At baseline, this cluster had the worst somatization and depression scores, and was least likely to be very satisfied with their life.

### 3.6 Stomach pain trajectories

Analysis of the stomach pain data using LCGA identified a four cluster solution as being the optimal model (see Fig. 1d). The largest cluster (cluster 1) had very low probability of pain throughout follow-up, and contained 79% of the sample. The next most common pain cluster had decreasing probability of pain between baseline and 3-years (cluster 2, 9%); this cluster had moderately high levels of somatization and depression (Table 2). Cluster 3 (7%) had rising probability of pain during follow-up, and had moderately high mean somatization and depression scores. The final cluster (5%) had high probability of pain throughout follow-up (cluster 4), and had the highest proportion of females, the highest mean baseline somatization and depression scores, and the lowest levels of life satisfaction.

## 4 Cluster characteristics at 3-years and change over time

For all four pain sites, absolute somatization and depression scores were the lowest at both baseline and 3-year follow-up in cluster 1, the ‘no pain problem’ cluster (Table 3). For the majority of clusters, there was an improvement in somatization and depression scores during follow-up. The largest improvements in depression (27–29% reduction in mean scores) were seen in back pain cluster 2, facial pain cluster 3, and stomach pain cluster 2, which all had decreasing probability of pain during follow-up. The largest improvements in somatization (42% and 42% decrease) were in back pain cluster 6 and stomach pain cluster 4; these clusters had high probability of pain during follow-up. Exceptions to this trend were back pain cluster 3, which had probability of pain rising steeply after 18 months follow-up, and headache cluster 4 which had consistently high probability of pain; both of these had increasing depression scores (16% and 8% increase), and the back pain cluster also had an increasing mean somatization score (10% increase). The highest mean 3-year depression and somatization scores were recorded for facial pain cluster 4 (1.19 and 0.88, respectively), which was a small cluster with consistently high probability of pain.

BMI increased by around 12% on average from 11-14 years, with no clear trends for any of the individual trajectories or pain sites, and no large differences between trajectories. However there were statistically significant differences in 3-year mean BMI within the facial pain and stomach pain clusters (Table 3). The 3-year mean PDS score was lower in the no pain problem cluster than the painful clusters, although the change since baseline was larger. There were no clear patterns of pubertal development between the different painful trajectories.

## 5 Overlap between pain sites

The ‘no pain problem’ clusters included 75–90% of the sample at each pain site. Overall 56% (*n* = 750) were in the ‘no pain problem’ cluster for all four pain sites. These subjects had the lowest levels of somatization, depression and life satisfaction at both baseline and 3-year follow-up (Table 4), and less than half of them were female. Less than 2% of this group (1.9%) were classified with significant depressive symptoms, and less than 3% (2.8%) had significant somatization. Half of the subjects with one or more ‘painful’ trajectories (23% of the total sample, *n* = 305) had pain at only that site, with 11% of the sample (*n* = 152) having two ‘painful’ trajectories, 6% (*n* = 83) having three ‘painful’ trajectories and 3% of the sample (*n* = 39) reporting ‘painful’ trajectories at all four pain sites.

There were clusters of adolescents for each pain site with high probability of pain throughout follow-up, representing 1–5% of the sample. Combining this information reveals that 12% of the sample (*n* = 153) were in a ‘persistent pain’ cluster for at least one pain site. At baseline and 3-year follow-up these subjects had the worst levels of life satisfaction, depression and somatization (Table 4), and over three-quarters of them were female. The proportion classified with significant depressive or somatic symptoms was much higher than the group with no pain problem (13.2% and 22.9%, respectively). The majority of these (70%, 8% of the total sample, *n* = 107) had a ‘persistent pain’ trajectory at only one site, 2% of the sample (*n* = 32) had two ‘persistent pain’ trajectories, and 1% of the sample (*n* = 14) had ‘persistent pain’ trajectories at three or four pain sites. A further 32% of the sample (*n* = 426) had a non-persistent pain cluster at one or more pain site (and no persistent pain cluster); these adolescents generally had characteristics in-between the no pain problem and persistent pain cluster members, although they did have slightly higher levels of physical activity at baseline (Table 4).

Agreement between being in a ‘no pain problem’ or a ‘painful’ cluster was examined for all pain sites. Kappa values ranged from 0.24 for the agreement between facial pain and headache to 0.37 for the agreement between stomach pain and headache. These kappa values are above the level of chance (i.e. kappa = 0), and can be considered as indicating ‘fair’ agreement. When agreement between being in a ‘persistent pain’ cluster or not at each site was examined, agreement was only slight between facial pain and the other pain sites (Kappa 0.10–0.19), and was fair for agreement between other pain sites (Kappa 0.25–0.29).

## 6 Discussion

We have uncovered groups of adolescents with different trajectories of change over time for back pain, facial pain, headache and stomach pain. Overall, trajectories identified at the different pain sites were similar, and four cluster solutions with similar patterns were optimal for facial pain, headache and stomach pain. Half of the adolescents included had low probability of frequent pain at all four pain sites throughout the 3-year follow-up period, identified by ‘no pain problem’ trajectories. However, 44% had a ‘painful’ trajectory for at least one pain condition, and headache, back pain and stomach pain were common, with over 20% of subjects in a ‘painful’ trajectory. Headache was the most common site, with a quarter of the sample being classified into one of the ‘painful’ trajectories, and facial pain was the least common (10%). These figures mirror previous work [21,27], which suggests that rates of facial pain are low before 14 years whereas headache, abdominal pain and back pain are more common [13,14,36]. The current study adds to evidence from prevalence studies which measure occurrence a single time, as it highlights a substantial proportion of adolescents with fair probability of pain between the ages of 11 and 14 years. However, only a small proportion of subjects were identified with a ‘persistent pain’ trajectory: 1–5% of the total depending on the pain site. Although this is a small proportion for each condition, across the four pain sites, 12% of the sample had a ‘persistent pain’ trajectory for at least one condition. This group was predominantly female, with the highest levels of somatization and depression at the start and end of the study period, and were the least likely to be satisfied with their life. This group represents a substantial proportion of the general adolescent population, but has never been specifically identified before.

Each pain site had one or more trajectories where the probability of pain increased during follow-up. In studies of incidence, these may have been identified as incident cases during adolescence, but the identification of specific trajectories gives more detail and is potentially more reliable. LCGA, which allocates people to clusters on the basis of their probability of being in that cluster, describes cluster-specific fluctuations in the pain experience. Studies simply using baseline and follow-up points might miss fluctuations or misclassify people on the basis of short-term flare-ups. Using data throughout follow-up better represents the general course of pain, but also allocates people to a particular trajectory even if they have a few months with an abnormal pattern. In addition, this method enables identification of groups who develop pain at different ages, for example, one group had increasing probability of back pain from the age of 11, whereas another did not show the tendency towards back pain until around 18 months later.

Combining data from four different painful sites gives a better representation of the true population burden than studying individual conditions. Overlap between trajectory membership was higher than chance, but only modest, indicating that, at this age, only a minority experience more than one frequent pain condition. This is interesting, as there are consistencies in the baseline characteristics of the pain clusters across the different pain sites. With the exception of facial pain, where there are very small numbers with ‘persistent pain’, subjects in the ‘persistent pain’ clusters are more likely to be female. In addition, they have, on average, higher baseline somatization and depression scores than those in other clusters. Somatic symptoms and depression also predict pain onset e.g. [40] and persistence of chronic disabling pain in adults [38]. These findings are an important contribution to the debate on whether these symptoms are different manifestations of the same underlying condition, or whether they are separate conditions [42]. It is possible that the tendency towards one condition is determined during adolescence, and persists throughout adulthood, as prior experience of a specific pain condition is a known risk factor for experiencing it again [16,23]. While this study included four common painful conditions, other pain sites, such as limb pain which can be common in adolescents [29], may be important. Further studies may wish to investigate pain trajectories at other sites.

This study uniquely presents frequently collected data from four pain sites on a large, population-based cohort, and uses novel statistical techniques to identify clusters of individuals with different pain trajectories. However, despite the large sample size, low prevalence of some pain conditions (notably facial pain) results in small numbers in some clusters. This may mask differences among identified clusters by reducing precision of the estimates. Differences in patterns of some clusters identified (e.g. back pain cluster 5 and facial pain cluster 3, where probability of experiencing pain rises and then falls) may be a result of small sample size. It would have been interesting to conduct analyses of each pain problem separately for different subgroups, (e.g. males and females), but even in a cohort of this size there is insufficient power to carry out such analyses. The population included is similar in terms of race and ethnicity to the population of the Seattle metropolitan area, but some individuals included in the analysis had missing data at up to three time points. LCGA has advantages over other analysis techniques, as it allocates individuals to the cluster that they have the highest probability of belonging to, given the data that they do have, so the impact of limited missing data is minimised. The majority of included subjects had high probability of belonging to their allocated cluster, but a small group (up to 10%) were placed in their cluster with less certainty. However, goodness of fit is one criteria for deciding the ideal number of clusters in LCGA, and overall the subjects within each cluster fitted the cluster they were in.

In addition to occasional missing data points for those included, approximately a third of subjects in the original sample were not included due to missing data at four or more data collection points. The non-included subjects tended to have higher scores on the psychological scales, and were more likely to have experienced pain previously. In addition, a significant predictor of adolescent participation was whether the parent reported child pain in the past 3 months [21]. Given this bias in initial response, and as history of pain and poorer psychological status are both risk factors for having pain conditions [20,23,24], the proportion of the cohort identified here with ‘no pain problem’ trajectories may have been over-estimated, and the proportion within ‘painful’ trajectories may be larger. While this may influence the sizes of clusters identified, it is difficult to imagine that it would substantially influence the trajectories identified, or the characteristics of those trajectories.

This paper fills gaps in current research, and also raises questions for future studies. First, our data provide new information on trajectories of pain among adolescents. This elucidates how pain conditions develop among adolescents, and also raises questions of how this fits into the experience of pain throughout the lifecourse. The ‘painful’ trajectories may represent the beginning of a lifelong tendency to experience pain, although further long-term research is needed to verify this, and if so, whether the experience of frequent pain in adolescence represents the beginning of a long-term condition at that specific site, or whether it is an early indication of a more general tendency to experience pain. Second, this research adds to the literature on whether symptom-based conditions such as pain are separate diseases, or different manifestations of a single condition. We have described consistency in trajectories identified at different pain sites, and similarities in subject characteristics within those clusters, but these trajectories generally have different people within them. It is unclear from our findings why some adolescents report back pain, for example, while others report facial pain. Further research in these two areas would add to the body of knowledge on pain experience over time and throughout the life course.

We have identified clusters of adolescents with different trajectories of back pain, facial pain, headache and stomach pain over time. Subjects with high probability of pain throughout the study period were more often female and had worse psychological status, regardless of the pain site studied. The experience of a ‘painful’ trajectory is relatively common, and if these individuals can be identified clinically, targeted interventions aimed at modifying persistent pain may be appropriate. The similarity in trajectories and characteristics of the clusters identified for the four pain conditions has implications for research and potentially for clinical practice. For example, the same research questionnaires can be used to measure the impact of each of these conditions, and certain types of interventions could be standardised across the pain sites. These findings provide new and valuable information, using a large cohort, with frequent follow-up, and novel statistical analyses, on the course of pain among adolescents.

## 7 Conflict of interest statement

None of the authors has any conflict of interest with this work.

## Figures and Tables

**Table 1:** Goodness of fit statistics for models of pain trajectory from latent class growth analysis.

Model	AIC_LL_	BIC_LL_	CAIC_LL_	Test of improvement in model fit compared to model with 1 less cluster^a^
*Back pain*				
3 Cluster	7033	7090	7101	<0.001
4 Cluster	6947	7025	7040	<0.001
5 Cluster	6917	**7016**	**7035**	<0.001
6 Cluster	6905	7025	7048	<0.001
7 Cluster	**6900**	7040	7067	**0.01**
8 Cluster	6900	7061	7092	0.19

*Facial pain*				
2 Cluster	4314	4351	4358	<0.001
3 Cluster	4277	4334	4345	<0.001
4 Cluster	4251	**4329**	**4344**	<0.001
5 Cluster	**4246**	4345	4364	**0.008**
6 Cluster	4252	4372	4395	0.58

*Headache*				
2 Cluster	7616	7652	7659	<0.001
3 Cluster	7340	7397	7408	<0.001
4 Cluster	7288	**7366**	**7381**	<0.001
5 Cluster	7273	7372	7391	<0.001
6 Cluster	7256	7376	7399	<0.001

*Stomach pain*				
2 Cluster	7003	7039	7046	<0.001
3 Cluster	6885	6943	6954	<0.001
4 Cluster	6834	**6912**	**6927**	<0.001
5 Cluster	**6822**	6921	6940	**<0.001**
6 Cluster	6822	6942	6965	0.17

AIC, Akaike’s information criterion; BIC, Bayes’ information criteria; CAIC, consistent Akaike’s information criterion; LL, log-likelihood. Optimal models based on goodness of fit statistics shown in bold.

a Bootstrap *p*-value.

**Table 2:** Baseline characteristics of clusters.

	*N* (% of total)	Female	Any strenuous physical activity last year	Very satisfied with life	Mean somatization score	Mean depression score	Mean BMI	Mean PDS score
*Back pain clusters*								
1	1045 (78%)	50.1%	82.2%	42.1%	0.40	0.61	19.52	2.02
2	138 (10%)	54.3%	87.0%	29.0%	0.75	0.88	19.46	2.17
3	54 (4%)	63.0%	88.9%	41.5%	0.52	0.72	18.94	2.25
4	47 (4%)	83.0%	78.7%	34.0%	0.86	0.95	19.44	2.24
5	31 (2%)	64.5%	87.1%	58.1%	0.47	0.66	19.95	2.07
6	18 (1%)	83.3%	88.9%	11.1%	0.96	1.09	19.70	2.42
*p-value*		<0.001	0.44	0.002	<0.001	<0.001	0.91	<0.001

*Facial pain clusters*								
1	1201 (90%)	52.5%	82.8%	41.6%	0.43	0.62	19.39	2.04
2	82 (6%)	48.8%	86.6%	29.3%	0.75	0.90	20.51	2.29
3	33 (2%)	75.8%	81.8%	30.3%	0.69	0.99	18.89	2.12
4	19 (1%)	63.2%	78.9%	21.1%	1.00	1.25	18.86	2.02
*p-value*		0.04	0.80	0.03	<0.001	<0.001	0.10	0.001

*Headache clusters*								
1	1007 (75%)	48.9%	81.9%	43.8%	0.39	0.59	19.32	2.02
2	146 (11%)	54.8%	85.6%	31.0%	0.70	0.90	19.73	2.13
3	113 (8%)	72.6%	89.4%	31.9%	0.62	0.84	20.23	2.29
4	69 (5%)	78.3%	82.6%	23.2%	0.80	0.99	19.19	2.17
*p-value*		<0.001	0.18	<0.001	<0.001	<0.001	0.10	<0.001

*Stomach pain clusters*								
1	1058 (79%)	49.8%	81.9%	42.3%	0.40	0.59	19.41	2.03
2	123 (9%)	61.0%	87.0%	33.6%	0.67	0.90	19.67	2.17
3	88 (7%)	64.8%	87.5%	39.1%	0.66	0.87	19.68	2.18
4	67 (5%)	74.6%	86.6%	25.4%	0.95	1.12	18.98	2.12
*p-value*		<0.001	0.25	0.02	<0.001	<0.001	0.82	0.009

Total sample	1337	53.0%	83.0%	40.4%	0.47	0.66	19.51	2.06

BMI, Body Mass Index; PDS, Pubertal Development Scale.

**Table 3:** Characteristics of clusters at 3 year follow-up and change from baseline.

	Somatization		Depression		BMI		Pubertal Development Scale	
	Mean score	% change from baseline	Mean score	% change from baseline	Mean	% change from baseline	Mean	% change from baseline
*Back pain clusters*								
1	0.29	−27%	0.45	−25%	21.66	11%	3.04	50%
2	0.50	−35%	0.65	−27%	21.79	12%	3.12	46%
3	0.58	10%	0.84	16%	23.11	22%	3.13	40%
4	0.68	−28%	1.01	−3%	22.18	14%	3.34	49%
5	0.32	−31%	0.58	−13%	22.38	12%	3.12	54%
6	0.53	−45%	1.01	−8%	21.42	9%	3.26	34%
*p-value*	<0.001		<0.001		0.162		0.009	

*Facial pain clusters*								
1	0.32	−27%	0.48	−22%	21.69	12%	3.07	50%
2	0.51	−31%	0.77	−14%	22.99	12%	3.10	35%
3	0.43	−38%	0.70	−29%	21.49	14%	3.19	49%
4	0.88	−12%	1.19	−5%	22.44	19%	3.31	64%
*p-value*	<0.001		<0.001		0.008		0.172	

*Headache clusters*								
1	0.29	−27%	0.44	−25%	21.63	12%	3.03	50%
2	0.44	−38%	0.69	−24%	21.76	10%	3.12	47%
3	0.51	−17%	0.69	−17%	22.67	12%	3.30	43%
4	0.64	−20%	1.07	8%	22.41	17%	3.20	49%
*p-value*	<0.001		<0.001		0.062		<0.001	

*Stomach pain clusters*								
1	0.29	−27%	0.46	−22%	21.64	11%	3.05	50%
2	0.48	−28%	0.65	−28%	22.67	15%	3.21	46%
3	0.59	−10%	0.78	−11%	22.24	13%	3.17	44%
4	0.55	−42%	0.91	−19%	21.52	13%	3.12	47%
*p-value*	<0.001		<0.001		0.018		0.01	

Total sample	0.34	−27%	0.53	−21%	21.78	12%	3.07	49%

BMI, Body Mass Index.

**Table 4:** Comparison of subjects with no pain, non-persistent pain or persistent pain at any site.

	No pain cluster at any site (*n* = 750)	Non-persistent pain cluster at one or more pain site (*n* = 426)	Persistent pain cluster at any site (*n* = 153)	*p-value*
Female	45.6%	57.7%	76.5%	<0.001

*Baseline*				
Very satisfied with life	45.0%	36.9%	26.1%	<0.001
Mean somatization score	0.35	0.55	0.84	<0.001
Mean depression score	0.54	0.78	0.96	<0.001
Mean BMI	19.42	19.67	19.50	0.58
Any strenuous physical activity last year	80.7%	87.3%	81.7%	0.014

*3-year follow-up*				
Very satisfied with life	42.2%	33.1%	26.9%	<0.001
Mean somatization score	0.25	0.42	0.58	<0.001
Mean depression score	0.39	0.61	0.91	<0.001
Mean BMI	21.50	22.12	22.14	0.025

*N* = 1329 subjects with trajectories identified at all four sites included. BMI, Body Mass Index.
